# Identification of COVID-19-Associated DNA Methylation Variations by Integrating Methylation Array and scRNA-Seq Data at Cell-Type Resolution

**DOI:** 10.3390/genes13071109

**Published:** 2022-06-21

**Authors:** Guoliang Wang, Zhuang Xiong, Fei Yang, Xinchang Zheng, Wenting Zong, Rujiao Li, Yiming Bao

**Affiliations:** 1 National Genomics Data Center, Beijing Institute of Genomics, Chinese Academy of Sciences and China National Center for Bioinformation, Beijing 100101, China; wangguoliang@big.ac.cn (G.W.); xiongzhuang17m@big.ac.cn (Z.X.); yangfei@big.ac.cn (F.Y.); zhengxinchang@big.ac.cn (X.Z.); zongwenting2018m@big.ac.cn (W.Z.); 2 CAS Key Laboratory of Genome Sciences and Information, Beijing Institute of Genomics, Chinese Academy of Sciences and China National Center for Bioinformation, Beijing 100101, China; 3 University of Chinese Academy of Sciences, Beijing 100049, China

**Keywords:** COVID-19, DNA methylation, methylation array, scRNA-seq, DNAm variation gene

## Abstract

Single-cell transcriptome studies have revealed immune dysfunction in COVID-19 patients, including lymphopenia, T cell exhaustion, and increased levels of pro-inflammatory cytokines, while DNA methylation plays an important role in the regulation of immune response and inflammatory response. The specific cell types of immune responses regulated by DNA methylation in COVID-19 patients will be better understood by exploring the COVID-19 DNA methylation variation at the cell-type level. Here, we developed an analytical pipeline to explore single-cell DNA methylation variations in COVID-19 patients by transferring bulk-tissue-level knowledge to the single-cell level. We discovered that the methylation variations in the whole blood of COVID-19 patients showed significant cell-type specificity with remarkable enrichment in gamma-delta T cells and presented a phenomenon of hypermethylation and low expression. Furthermore, we identified five genes whose methylation variations were associated with several cell types. Among them, *S100A9*, *AHNAK*, and *CX3CR1* have been reported as potential COVID-19 biomarkers previously, and the others (*TRAF3IP3* and *LFNG*) are closely associated with the immune and virus-related signaling pathways. We propose that they might serve as potential epigenetic biomarkers for COVID-19 and could play roles in important biological processes such as the immune response and antiviral activity.

## 1. Introduction

Severe acute respiratory syndrome coronavirus 2 (SARS-CoV-2) infection has spread rapidly around the world, resulting in the COVID-19 pandemic [1]. The clinical manifestations of SARS-CoV-2 infection have great differences, ranging from asymptomatic to life-threatening [2]. A series of single-cell transcriptome studies on COVID-19 have been conducted, which have made significant contributions to COVID-19 diagnosis, prevention, and treatment. Immune imbalance has been reported in COVID-19 patients, and the disorders of innate and adaptive immune responses can lead to a more severe course of disease [3,4]. Monocytes and NK cells in COVID-19 patients show significantly up-regulated IFN reactions and acute inflammatory responses, as well as increased apoptosis and migration levels [5]. Epigenetics opens the door to host-pathogen interactions that can reveal the biological relationship between host and pathogen [6]. At present, numerous epigenetics studies have revealed a significant relationship between DNA methylation and immune system steady-state and inflammatory response. The *IFITM3* gene, which regulates the expression of the antiviral interferon-induced transmembrane protein 3, is selectively demethylated in memory CD8 T cells in the lung during influenza infection in mice [7]. The de novo DNA methyltransferases Dnmt3a and Dnmt3b are genetically deleted in B cells, resulting in normal B cell growth and maturation in mice [8]. There is a distinct DNA methylation (DNAm) feature of COVID-19 patients where methylation levels of interferon-related genes are elevated and methylation levels of inflammatory genes are decreased [6]. Significant overrepresentation of binding sites of the CCAAT Enhancer Binding Protein beta(*CEBPB*) in hypomethylated regions affects emergent granulopoiesis and B-lymphocyte-to-granulocyte trans-differentiation [9]. Differentially methylated genes in B cells, T cells, macrophages and neutrophils are enriched in antiviral activity, cytokine production, inflammation, and innate and adaptive immunity [10].

At present, the bulk-tissue DNA methylation study has made significant progress on COVID-19. However, the bulk-tissue DNA methylation level is an average of all cell types in the tissue, which limits our understanding of the contributions of specific cell types to DNA methylation variations [11]. Single-cell DNA methylation sequencing makes it possible to explore cellular epigenetic heterogeneity [12]. Unfortunately, it is unscalable to large cohort studies because the technologies are costly and not yet mature [13,14,15]. Bulk-tissue studies in the past ten years have resulted in the accumulation of massive data and corresponding phenotype information [16,17,18]. Hence, we propose a new analytical pipeline using DNA methylation array data at the bulk level, scRNA-seq data, and the regulatory relationship between methylation and expression, trying to transfer bulk-tissue phenomena to the single-cell DNA methylation level to reveal specific cell types of DNA methylation variations in COVID-19. This will facilitate the discovery of potential COVID-19 DNA methylation biomarkers to help in the diagnosis of COVID-19. At the same time, our research can find targeted cells for drug treatment, which has a strong guiding role in the design of drugs for COVID-19.

## 2. Materials and Methods

### 2.1. The Construction of the Pipeline

**Figure 1:**
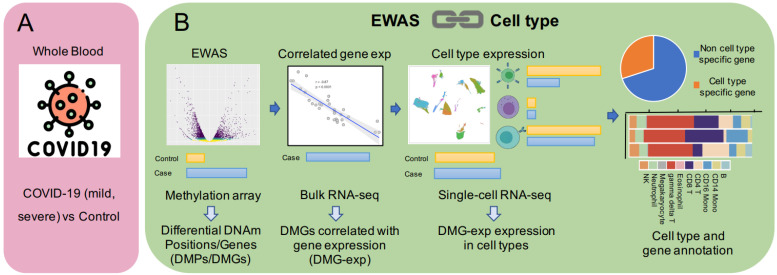
(**A**): All datasets are from whole blood, whereas the scRNA-seq dataset contains mild and severe COVID-19 samples. (**B**): The pipeline contains three parts: differential methylation analysis, DNAm-mRNA correlation analysis, and single-cell RNA-seq data analysis.

The pipeline consisted of three parts: (1) Differential methylation analysis. Firstly, differential methylation analysis was performed on a trait to obtain differentially methylated positions and genes (DMPs/DMGs), which was also a common analysis strategy for epigenome-wide association studies (EWAS) [19,20]. (2) Calculating the correlation between gene methylation and expression. Since DNA methylation, as one of the important epigenetic regulatory mechanisms, has a stable regulatory relationship with gene expression in terms of tissue and cells [21,22], the correlation matrix between the methylation position and annotated gene was generated using the matched DNA methylation and gene expression data in the same patients. DMGs correlated with gene expression (DMG-exp) were selected for further analysis based on correlation and significance. (3) Single-cell level analysis. Differential expression analysis was performed in cell clusters in single-cell datasets to identify differentially expressed genes and their related cell types via comprehensive and accurate cell annotation (SingleR [23]). The genes obtained above were further annotated to specific cell types to obtain cell type-specific and non-cell-type-specific genes (Figure 1). It should be pointed out that while exploring a certain trait, the different omics data should be taken from the same tissue to ensure the validity of the conclusions.

### 2.2. Data Collection

Data for analysis were taken from the GEO and EGA databases (Table 1). The MethylationEPIC BeadChip datasets (GSE174818 [10], GSE153712 [24]) were used to analyze differential methylation from whole blood between patients and healthy controls. Bulk-tissue DNAm (GSE174818) and matched mRNA datasets (GSE157103 [25]) were used for correlation analysis. The scRNA-seq datasets (GSE157344 [26], EGAS0000001004571 [27]) were used for single-cell transcriptome analysis. The two datasets both contain healthy controls and COVID-19 patients with mild or severe symptoms. GSE157344 was used as a discovery dataset and EGAS00001004571 as a validation dataset. The clinical status of the patients was defined according to the WHO ordinal scale.

### 2.3. Differential Methylation Analysis 

GMQN [28] and IMPUTE [29] were used to correct the batch effect and fill missing values. The sex chromosome and SNP-related probes were filtered before differential methylation analysis. CHAMP was used to identify differentially methylated sites (FDR < 10^−9^ with Welch’s *t*-test) between COVID-19 and control samples while considering the effects of age and gender [30].

### 2.4. DNAm-mRNA Correlation Analysis

Using a matched bulk-tissue DNAm-mRNA dataset and Infinium MethylationEPIC BeadChip annotation files, the DNAm-mRNA correlation matrix was calculated between the methylation levels (β value) of probes and the expression levels (TPM) of annotated genes. A Pearson correlation coefficient greater than 0.5 and a *p*-value less than 0.05 were defined as a strongly correlated probe-gene annotation with the chi-squared test.

### 2.5. Single-Cell RNA-Seq Data Analysis 

The 10x scRNA-seq UMI counts matrix was imported into R V4.0.5, and the expression data were analyzed with Seurat V4.0.5 [31]. Low-quality cells were removed based on the following criteria: less than 500 UMIs, more than 25% mitochondrial reads, less than 200 expressed genes or more than 6000 expressed genes. “NormalizeData” and “ScaleData” functions were used to normalize and scale each sample, respectively, and the “VST” method in Seurat was used to identify the 2000 most variable genes for principal component analysis (PCA). Then we applied the integrated methods in Seurat to eliminate batch effects between different samples (function “FindIntegrationAnchors” and “Integratedata”, dims = 1:30). After the UMAP calculation, the top 30 principal components with a resolution of one were chosen for clustering. Using the “FindAllMarkers” function in Seurat, we performed differential expression analysis based on the disease status of samples in each cluster. Genes having a *p*-value less than 0.01 and log-fold changes more than 0.2 were considered significantly differentially expressed with the Wilcoxon rank-sum test. This study was carried out on three data subsets: COVID-Control, Mild-Control, and Severe-Control. The “FindAllMarkers” function was also used to identify cluster marker genes by differential expression analysis between one cluster and others. Finally, cellular identity was determined by comparing cluster marker genes in the original dataset with known cell-type-specific genes and utilizing annotation tools from SingleR [23] and DISCO databases [32]. 

## 3. Results

### 3.1. DNA Methylation Variations in Whole Blood of COVID-19 at the Cell-Type Resolution

**Figure 2:**
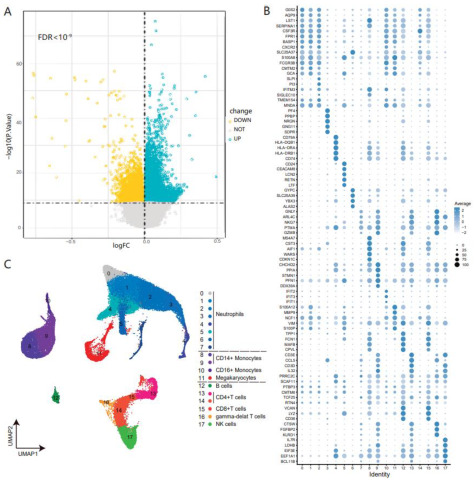

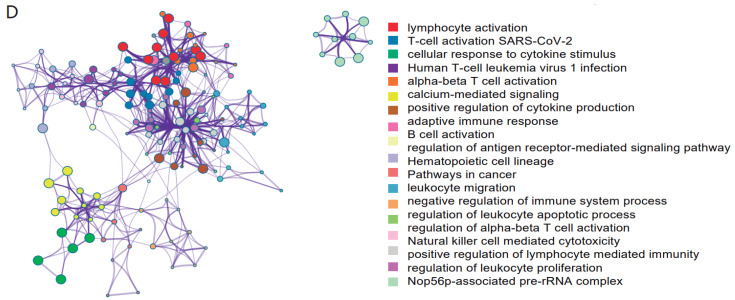
(**A**): Volcano plot of differential methylation analysis. (**B**): Dot plots of the top 5 marker genes form each cluster sorted by the average log fold change. (**C**): UMAP visualization of scRNA-seq data from 26 COVID-19 samples (6 mild, 20 severe COVID-19 patients) and 5 healthy controls colored according to different cell clusters. (**D**): Network plot of gene ontological biological processes related to 388 differentially methylated genes, ordered by statistical significance.

Using the pipeline described above, we performed an integrated analysis of methylation array data, RNA-seq data, and scRNA-seq data from the whole blood of COVID-19 patients and healthy controls. Firstly, we performed differential methylation analysis (see Section 2.3). A total of 21,025 differentially methylated positions were obtained, although average DNA methylation abundance across the whole genome did not significantly differ between COVID-19 patients and healthy controls (Figure 2A). Then, the DNAm-mRNA correlation was calculated using bulk DNA methylation with matched RNA-seq data of COVID-19 patients and healthy controls. Finally, after annotation and screening of differentially methylated positions, we obtained 388 differentially methylated genes of COVID-19 that were significantly correlated with expressions (see Section 2.4). According to the gene ontology enrichment analysis, the majority of the 388 genes are enriched in immune-related terms such as negative regulation of immune system process, positive regulation of lymphocyte-mediated immunity, and regulation of α-β T cell activation (Figure 2D). In particular, “T-cell activation SARS-CoV-2”, a GO term related to SARS-CoV-2, was significantly enriched (*p*-value < 10^−14^). For single-cell transcriptome analysis, cell types were annotated by combining annotation tools along with high-quality manual annotation (Figure 2B,C and Supplementary Figures S1 and S2). Moreover, differential expression analysis in each cell type showed that the expressions of 92 of the 388 genes were inconsistent in the different cell types (Supplementary Table S1). They were considered to be differentially expressed genes that were highly correlated with COVID-19-associated DNA methylation variations in our analysis.

### 3.2. Methylated Variant Genes Associated with COVID-19 Have Significant Cell-Type Specificity

**Figure 3:**
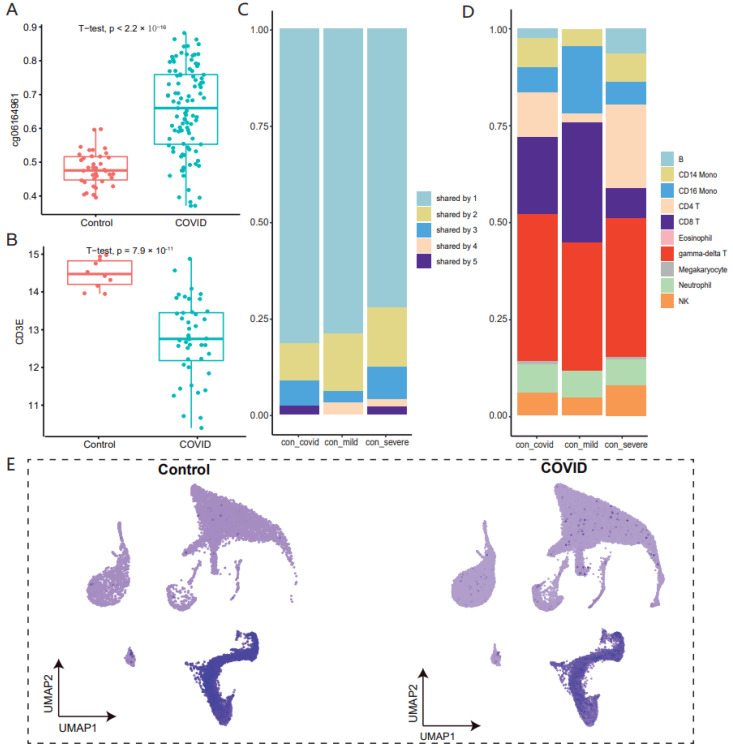

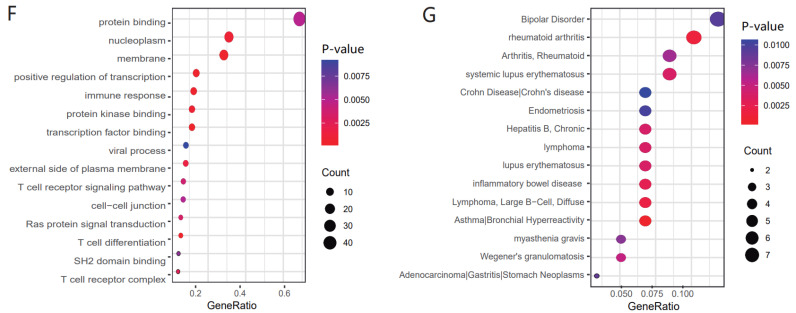
(**A**,**B**): A box-and-whisker plot depicts the difference in *CD3E* methylation (**A**) and expression (**B**) level (y-axis) between COVID-19 patients and healthy controls (x-axis). (**C**,**D**): The stacked bar chart shows the frequency of cell-type-related genes (**C**) and cell types (**D**) for control-COVID, control-mild and control-severe COVID-19 patients. (**E**): The feature plot depicts the difference in *CD3E* expression in single-cell transcriptome between COVID-19 patients and healthy controls. (**F**,**G**): Bar graph of gene ontological (GO) and disease ontological (DO) biological processes related to gamma-delta T cell-associated genes, ordered by statistical significance.

Our study showed that the methylation variations of these genes were associated with one or a few cell types rather than all cell types. About 70% of genes were cell type-specific in COVID-19 patients (Figure 3C). According to the gene ontology enrichment analysis, the majority of cell-specific genes were enriched in common processes characterizing COVID-19, such as transcription factor binding, immune response, T cell differentiation, and viral process (Figure 3F). Moreover, the disease ontology analysis revealed that these genes were enriched in asthma and other diseases caused by viruses, including viral hepatitis type B (Figure 3G). Furthermore, considering the difference between mild and severe symptoms, we also discovered that gene methylation variations were significantly cell-type-specific. In the validation dataset, consistent results were observed (Supplementary Figure S3). These findings suggest significant specificity of the methylated variant genes identified, as well as the strong correlation between COVID-19 infection and immunity.

### 3.3. The Majority of Methylated Variant Genes Are Associated with Gamma-Delta T Cells in COVID-19

Gamma-delta T cells are a distinctive T cell subpopulation, an important cell type to regulate immune response [33]. In most cases, responses of gamma-delta T cells are targeted at microbial pathogens [34]. At present, there are various cancers that use activated gamma-delta T cells for immunotherapy. The activation of gamma-delta T cells can induce the release of cytotoxic molecules and the secretion of cytokines, thereby inducing cancer apoptosis [35]. At the same time, gamma-delta T cells show excellent antiviral capacity in COVID-19 patients [36], according to single-cell transcriptome studies, while their pro-inflammatory activity can increase the risk of cytokine storm in COVID-19 patients [37]. In the whole blood of COVID-19 patients, we observed that 65% of the methylation variation genes were associated with T lymphocytes, and 50% of the genes were associated with gamma-delta T cells (Figure 3D). We subsequently examined changes in methylation and expression levels of these gamma-delta T-related genes in bulk-tissue methylomes and transcriptomes, finding that almost 90% of genes had higher methylation and decreased expression levels in COVID-19 patients, such as *CD3E* (Figure 3A,B). At the same time, we also found significantly low expression of *CD3E* in T cells and NK cells (Figure 3E). Similar trends were observed in the mild and severe symptom datasets, as well as the validation dataset (Supplementary Figure S4). We speculate that the increased methylation level of gamma-delta T cells in whole blood may lead to the decreased expression level in whole blood, thus participating in the immune process of COVID-19. COVID-19 causes a storm of inflammatory factors, so now the targeted cells for treatment focus on NK cells and granulocytes. We found that half of the DNA methylation variations genes we identified were associated with gamma-delta T cells, so gamma-delta T cells could be considered a new significant target cell when designing epigenetic drugs.

### 3.4. Multiple Cell-Type-Related Genes May Be Important Regulatory Genes of COVID-19 Immune Response

Five methylation variation genes involving more than four cell types were identified, including *S100A9*, *CX3CR1*, *LFNG*, *TRAF3IP3*, and *AHNAK*. Among them, *S100A9*, *AHNAK*, and *CX3CR1* have been reported to be important biomarkers for COVID-19. *S100A9*, a calcium-binding protein belonging to the *S100* family, is abundant in neutrophils [38]. It is the main alarmin molecule of the immune response system and can regulate inflammatory responses by recruiting immune cells and stimulating the secretion of certain cytokines [39]. The current study found that *S100A9* is a serological biomarker for risk assessment in COVID-19 patients [40]. As a member of the chemokine receptor superfamily, *CX3CR1* is mostly expressed on cytotoxic effector lymphocytes, such as natural killer cells (NK cells), cytotoxic T lymphocytes (CT lymphocytes), and macrophages. Additionally, it is a highly selective chemokine receptor and surface marker on cytotoxic effector lymphocytes [41]. The expression of *CX3CR1* may be associated with the pathological mechanism of severe COVID-19, and the low expression of *CX3CR1* in immune cells may contribute to the antiviral response [42]. As a scaffolding protein in many protein-protein interactions, *ANHAK* can coordinate a variety of biological processes, including tumor suppression and immune regulation [43]. *ANHAK* is the pro-viral factor for SARS-CoV-2 infection [44], while it is associated with COVID-19 in the progression of chronic obstructive pulmonary disease (COPD) [45]. Notably, we identified two genes (*TRAF3IP3* and *LFNG*) that have not been reported to be associated with COVID-19, which may be potential COVID-19 biomarkers or involved in the immune response to COVID-19. The SARS-CoV-2 M protein inhibits type I and III *IFN* production by targeting *RIG-I/MDA-5* signaling, which reduces antiviral immunity and increases viral replication. Tumor necrosis factor receptor-related factor 3 (*TRAF3*) interacting protein 3 *(TRAF3IP3)* plays a role in the development of immunological tissues as well as the immune response of the body [46]. *TRAF3IP3* is a critical regulator for the downstream *RIG-I/MDA-5* signaling pathway, and it accumulates on mitochondria in response to viral infection, driving *TRAF3* to *MAVS* for *TBK1-IRF3* activation [47]. Lunatic Fringe (*LFNG*) is a critical gene in the Notch signaling pathway that encodes the glycosyltransferase that may detect and glycosylate epithelial growth factor (*EGF*) repeats on Notch receptors, contributing to the regulation of disease onset and development. [48]. In addition, the Notch signaling pathway can interact with many viral particles, thereby promoting viral particles’ infectivity, and it is also involved in the inflammatory response [49]. 

## 4. Discussion

The single-cell transcriptome identifies changes in immune cell composition and activation of COVID-19 patients. T cells are decreased and exhausted, and neutrophils are increased significantly [27]. Monocytes and NK cells show significantly up-regulated IFN reactions and acute inflammatory responses, as well as increased apoptosis and migration levels [5]. DNA methylation plays key roles in immune system homeostasis and inflammatory responses [50]. However, due to the current limitations of single-cell methylation sequencing technology and the cost [13], studies of DNA methylation in COVID-19 patients were only carried out at the bulk-tissue level, which may not explain cell-type-specific DNA methylation variations. We developed an effective new analytical pipeline that attempts to transfer bulk-tissue phenomena to the single-cell DNA methylation dimension to reveal specific cell types of DNA methylation variations in COVID-19 by integrating multi-omics data. As a result, 92 genes were identified, and we found that the differential methylation of these genes was associated with one or several cell types rather than all cell types.

Cell-type-specific DNA methylation variations are found in around 70% of these genes. Most cell-type-specific genes were enriched in immune-related functions, as well as asthma and other diseases caused by viruses, according to gene ontology and disease ontology analyses. Meanwhile, we discovered that DNA methylation variance in COVID-19 patients was predominantly detected in gamma-delta T cells. Analysis of bulk tissue methylomes and transcriptomes revealed a tendency of increasing methylation and reduced expression for these genes. We, therefore, believe that increased methylation of T cells in whole blood may lead to lower gene expression, thus contributing to COVID-19’s immunological process.

In addition, we found five genes, namely *S100A9*, *CX3CR1*, *LFNG*, *TRAF3IP3*, and *AHNAK*, whose methylation variations were linked to more than four cell types. *S100A9*, *AHNAK*, and *CX3CR1* have all been identified as relevant COVID-19 biomarkers. We speculate that *TRAF3IP3* and *LFNG* methylation variations may serve as potential epigenetic biomarkers for COVID-19, and that they play a role in important biological processes like the immune response and antiviral activity [47,48]. 

We believe that our findings may help with COVID-19 diagnosis and prognosis. Currently, the clinical diagnosis of COVID-19 is based on chest imaging and routine blood leukocyte counts. The methylation level of gamma-delta T cells in the blood can be used to enhance clinical diagnosis, according to our findings. Because of the inflammatory storm induced by COVID-19, the targeted cells for treatment are now NK cells and granulocytes. We discovered that gamma-delta T cells were related to half of the DNA methylation variation genes we identified, suggesting that gamma-delta T cells could be a potential key target cell for developing epigenetic medicines.

## 5. Conclusions

We constructed the analysis pipeline to identify DNA methylation variations by integrating methylation array and scRNA-seq data at the cell-type resolution. Most of the 92 genes we identified were cell type-specific, and DNA methylation variations of these genes were concentrated in gamma-delta T cells, which may be the most efficient target cells for COVID-19 drug treatment. At the same time, we found five important genes whose DNA methylation variation may occur in multiple cells. Following confirmation in the literature and associated pathway analyses, we suggest that these may be valuable COVID-19 biomarkers that can aid in clinical diagnosis, prognosis, and therapy.

## 6. Limitations of the Study

This study attempts to exploit the strong correlation between gene methylation and expression to annotate methylation variation genes in tissues into single cells, thereby investigating the cell types that affect gene methylation variation at the single-cell level. We found that the methylation variations of the genes are cell type-specific, and the conclusions of the two sets of data are comparable, but further validation relying on single-cell methylation data is needed. Secondly, due to the sample heterogeneity between bulk-tissue and single cells, only 92 differentially methylated genes were eventually identified, potentially filtering out a large number of genes. Finally, we identified five potential epigenetic marker genes for COVID-19, but further verification by biological experiments is required.

## Figures and Tables

**Table 1:** Overview of datasets in the analysis.

Accession	Samples	Type	Platform	Annotation
GSE153712	38	Control	EPIC	Bulk-tissue
GSE174818	101	COVID-19	EPIC	Bulk-tissue
GSE157103	101	COVID-19	RNA-seq	Bulk-tissue
GSE157344	31	Control; Mild; Severe	10× scRNA-seq	Single-cell
EGAS0000001004571	16	Control; Mild; Severe	10× scRNA-seq	Single-cell

## Data Availability

Data reported in this paper are available from the GEO database, EGA database and EWAS Open Platform.

## Supplementary Materials

The following are available online at https://www.mdpi.com/article/10.3390/genes13071109/s1, Figure S1: Dot plots of the top 5 marker genes form each cluster sorted by the average log fold change, Figure S2: UMAP visualization of scRNA-seq data from 26 COVID-19 samples (8 mild, 10 severe patients) and 22 healthy controls colored according to different cell clusters, Figure S3: The stacked bar chart shows the frequency of cell-type-related genes for control-COVID, control-mild and control-severe COVID-19 patients, Figure S4: The stacked bar chart shows the frequency of cell types for control-COVID, control-mild and control-severe COVID-19 patients. Table S1: Differentially methylated genes with significantly correlation between DNAm with expression and Methylated variant gene annotation.
