# Supplementary figures and images for: Identification of COVID-19-Associated DNA Methylation Variations by Integrating Methylation Array and scRNA-Seq Data at Cell-Type Resolution
Source: Genes (Basel). 2022 Jun 21;13(7):1109. doi: 10.3390/genes13071109 (PMC9322889; doi:10.3390/genes13071109)

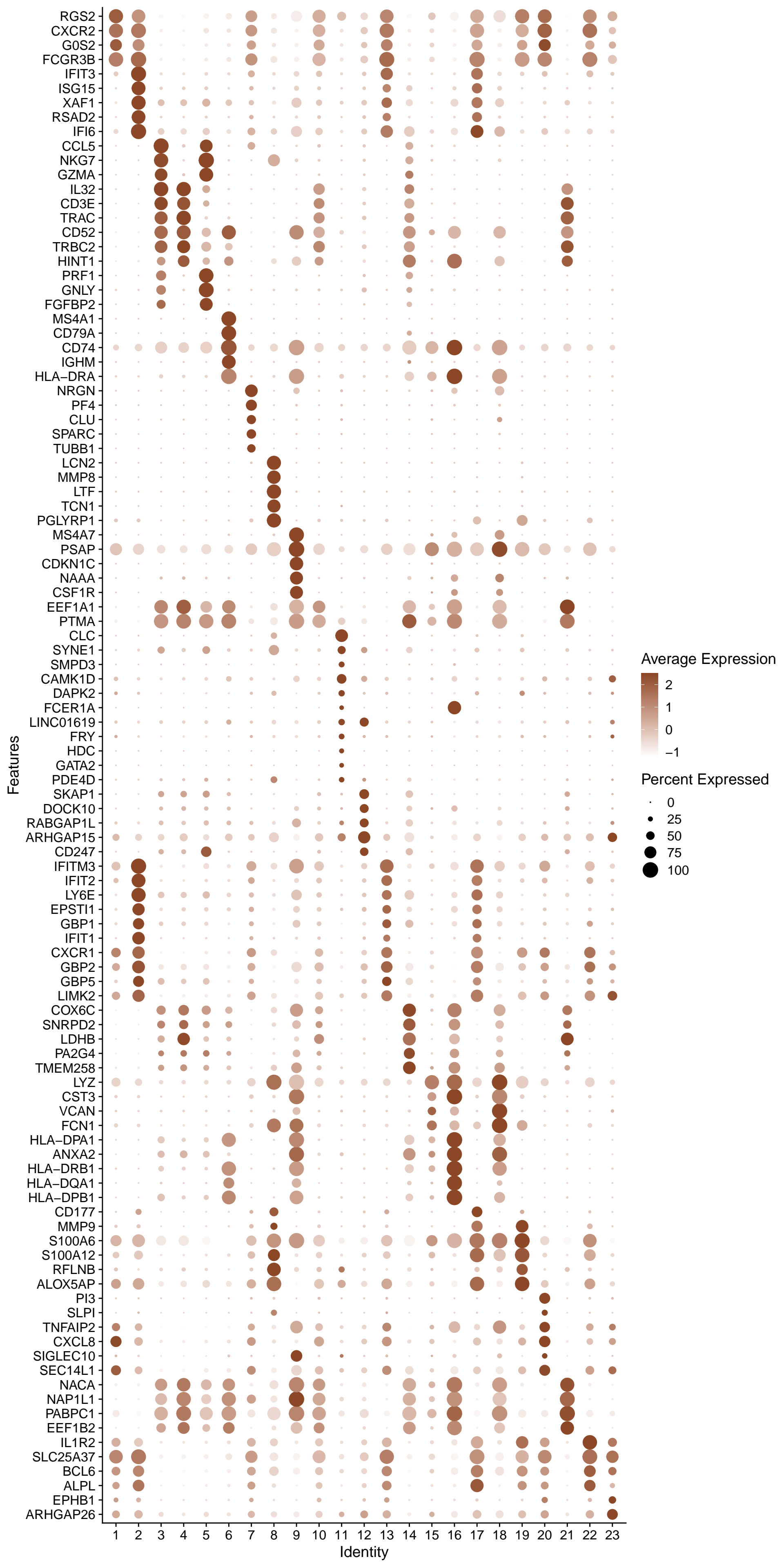

Supplement: Supplementary file 1 [file genes-13-01109-s001.zip › Supplementary Figure S1.pdf]

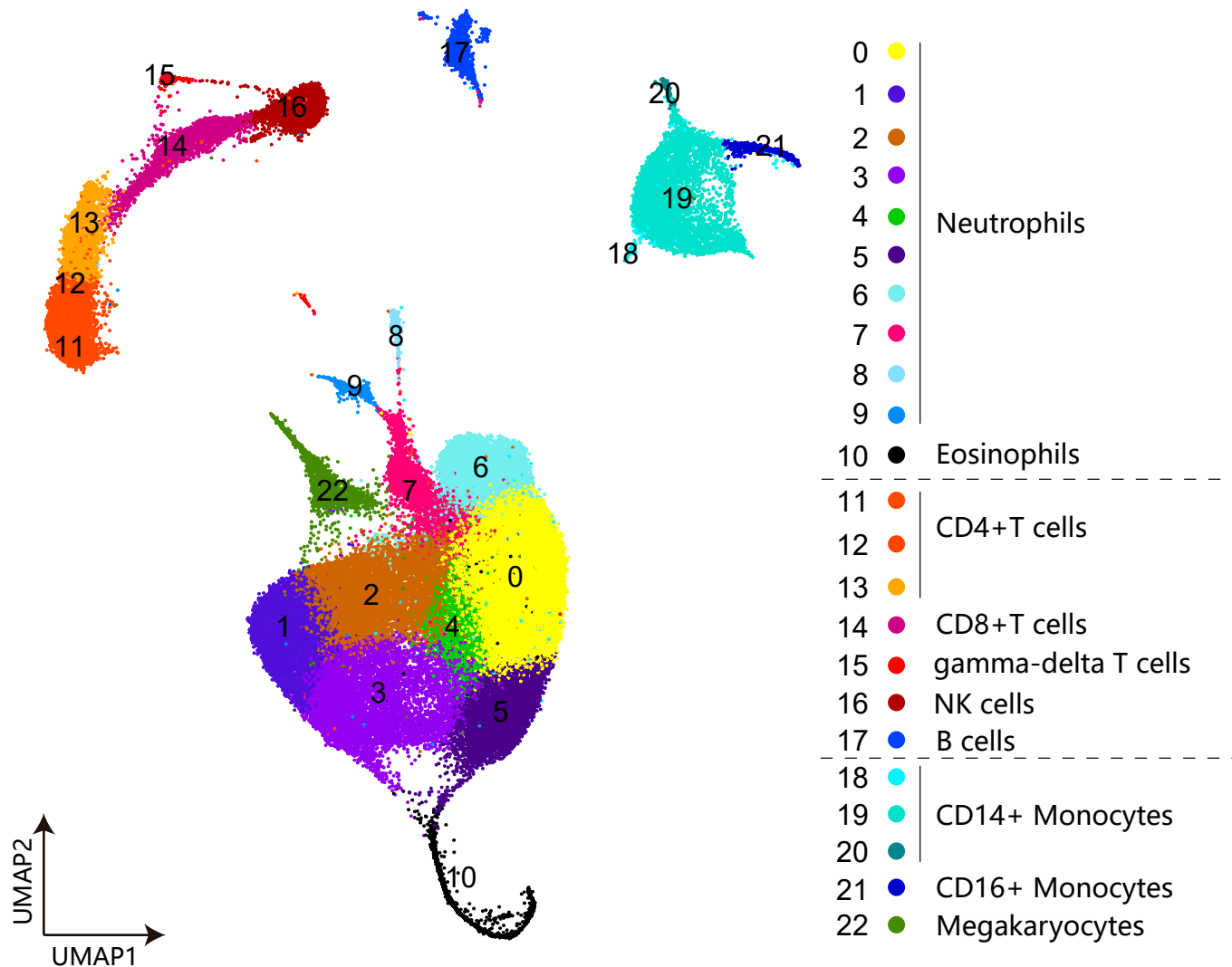

Supplement: Supplementary file 1 [file genes-13-01109-s001.zip › Supplementary Figure S2.pdf]

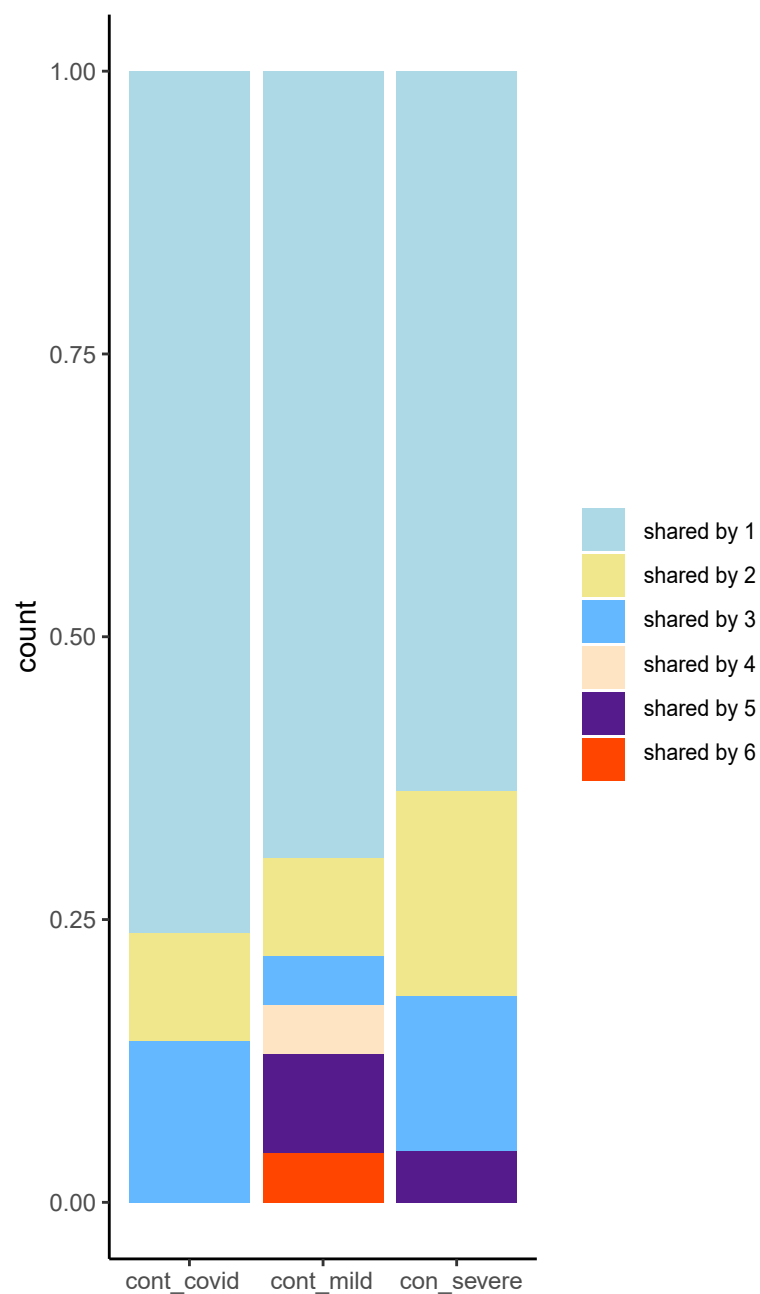

Supplement: Supplementary file 1 [file genes-13-01109-s001.zip › Supplementary Figure S3.pdf]

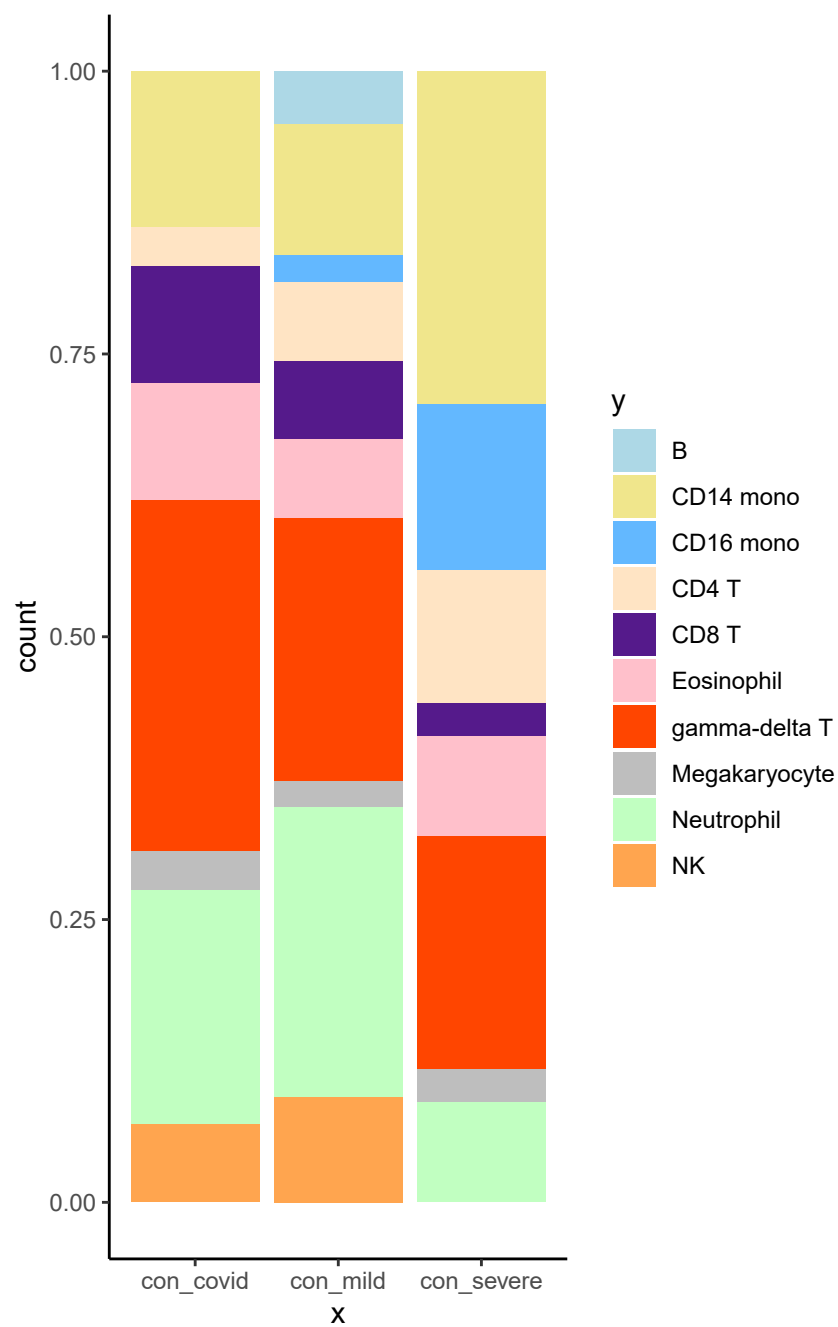

Supplement: Supplementary file 1 [file genes-13-01109-s001.zip › Supplementary Figure S4.pdf]
